# Eco-Efficiency of the English and Welsh Water Companies: A Cross Performance Assessment

**DOI:** 10.3390/ijerph18062831

**Published:** 2021-03-10

**Authors:** Ramon Sala-Garrido, Manuel Mocholi-Arce, Maria Molinos-Senante, Michail Smyrnakis, Alexandros Maziotis

**Affiliations:** 1 Department of Mathematics for Economics, University of Valencia, 46010 Valencia, Spain; Ramon.Sala@uv.es (R.S.-G.); manuel.mocholi@uv.es (M.M.-A.); 2 Departamento de Ingeniería Hidráulica y Ambiental, Pontificia Universidad Católica de Chile, 4860 Santiago, Chile; mmolinos@uc.cl; 3 Hartree Centre, Science and Technology Facility Council (STFC), Daresbury, Warrington WA4 4AD, UK; m.smyrnakis@gmail.com

**Keywords:** cross eco-efficiency, greenhouse gas emissions, environmental variables, water utilities, England and Wales

## Abstract

Analyzing costs and greenhouse gas (GHG) emissions could be of great importance for the water utilities to supply water services in a healthy and sustainable manner. In this study, we measured the eco-efficiency of several water utilities in England and Wales by incorporating GHG as an undesirable output. For the first time, we evaluated the eco-efficiency of the water production process using robust cross-efficiency data envelopment analysis (DEA) techniques. The further use of clustering and regression techniques allowed us to better understand the drivers of eco-efficiency. The results showed that the mean eco-efficiency of the water sector was 0.748, which indicates that costs and GHG emissions could be reduced by 25.2% to generate the same level of output. Large water companies with high energy costs and levels of GHG emissions belonged to the less eco-efficient group. Environmental factors related to density, topography, and treatment complexity further impacted eco-efficiency. Finally, we linked our results to the regulatory cycle and discuss some policy implications.

## 1. Introduction

Water and energy are two resources that are highly linked and will influence the future of the economy and environment. Energy is used to abstract and treat raw water, and subsequently to treat collected wastewater before it is safely and healthy discharged to the environment. The activities to provide water and wastewater services result in the release of greenhouse gas (GHG) emissions in the atmosphere which harm the health of people and environment [1]. Greenhouse gas emissions in the water and sewerage industry embrace scope 1, scope 2 and scope 3 emissions. It is estimated that 1%–2% of the total global primary energy use and 6% of regional electricity usage is associated with the water industry [2,3,4].

Therefore, reducing energy costs and GHG emissions, and moving to a sustainable urban water cycle, is a priority for water utilities [5]. Wakeel et al. [6] studied the use of energy in the production of drinking water and wastewater treatment at a country level. The authors found that energy intensive technologies used to treat water and wastewater could lead to high levels of GHG emissions. Other studies by Chen et al. [7] and Liao et al. [8] conducted at a city level showed that energy demand could substantially increase in the water cycle due to population growth and climate change. As a result, policy makers, academics, and professionals have been working towards the implementation of a net zero carbon water industry. For instance, the state of Victoria in Australia aims to obtain net zero carbon emissions in the water industry by 2050 [9]. In an analogous manner, the United Kingdom (UK) government aims to cut down its GHG emissions in the water sector by 80% by 2050 [4]. A greater understanding of both economic (energy and other costs) and environmental (GHG emissions) efficiency is of great importance for the water and sewerage sector to deliver its services in a sustainable and healthy way.

From a methodological point of view, the evaluation of water utilities’ efficiency can be conducted using parametric (econometric, e.g., stochastic frontier analysis (SFA)) and non-parametric (linear programming, e.g., data envelopment analysis (DEA) techniques). Unlike parametric approaches, non-parametric techniques do not require the specification of a functional form for the underlying technology [10]. Among non-parametric techniques, DEA has been widely used to compare each company’s efficiency relative to the “industry best practice” frontier [11,12]. Therefore, this study used DEA techniques to evaluate water utilities’ eco-efficiency. Eco-efficiency is defined as the production of more goods (outputs) and services with fewer resources (inputs) and a smaller environmental impact. The prefix ‘eco’ represents the environmental and economic performance; therefore, the assessment of eco-efficiency involves considering both environmental and economic variables. Few previous studies have measured the performance of water utilities by integrating GHG emissions as an undesirable output. Ananda and Hampf [13] and Ananda [9,11] used DEA techniques to estimate eco-efficiency in several water companies in Australia. The limitation of the above studies is that eco-efficiency was estimated using traditional DEA techniques. This means that the eco-efficiency of each water company (decision-making unit—DMU) was estimated based on a self-evaluation framework which might lead to eco-efficiency scores being over estimated [14]. This is due to the fact that the weights of the variables used in the analysis could take zero values and, therefore, these observations would not be part of the evaluation process. To overcome this limitation, Liu et al. [15] imposed restrictions on weights that prevented them from taking zero values and used cross-efficiency techniques. By doing this, the authors ensured that all observations were included in the evaluation. Moreover, each DMU was required not only to be self-evaluated but also to be peer-evaluated [16,17,18]. However, the main limitation of the cross-efficiency technique is that weights are not optimal [16]. To address this issue, two main approaches were developed by Sexton et al. [19] and Doyle and Green [16]. The first is the aggressive cross-DEA approach, which maximizes the efficiency of each DMU when the efficiency of the other DMUs is minimized [19]. The second approach is the benevolent cross-DEA approach, which maximizes the efficiency of each DMU when the efficiency of the other DMUs is maximized [16]. Liu et al. [15] developed an approach that included both the aggressive cross-DEA model and companies’ best ranking positions.

Cross-efficiency techniques have been used to evaluate eco-efficiency integrating undesirable outputs (e.g., GHG emissions) in several sectors such as energy [14,15], airlines [20], and agriculture [21]. However, to the best our knowledge, no prior studies in the water industry have applied cross-efficiency techniques with undesirable outputs to estimate the eco-efficiency of water utilities. Our study aims to fill this gap in the literature. Additionally, the model developed by Liu et al. [15], which considers restrictions in weights and includes firms’ intentions of pursuing the best ranking positions, has previously been applied in coal-fired power plants. To the best of our knowledge, no previous applications to the water industry have been made in England and Wales or elsewhere. Therefore, in this study we first assessed the eco-efficiency of water companies without imposing any restrictions on weights for desirable and undesirable outputs. To improve the accuracy of efficiency scores, we imposed restrictions on weights. This evaluation was based on self and peer evaluation of the units involved in the analysis, i.e., cross-efficiency techniques. Finally, to improve the robustness of efficiency scores, we evaluated the eco-efficiency of firms by including their intention of pursuing the best ranking positions. This evaluation was conducted using cross-efficiency techniques. 

Against this background, the main objective of this study was to estimate the eco-efficiency of several water utilities using cross-efficiency techniques. The robustness of each eco-efficiency technique was further analyzed and discussed. Furthermore, to obtain a greater understanding of water utilities’ performance, we employed cluster analysis to analyze and group water companies with similar characteristics based on their eco-efficiency scores. The last step of our analysis was to employ econometric techniques to understand whether variables in addition to costs could significantly impact companies’ eco-performance. These variables were related to topography, water treatment complexity, and density.

We make the following contributions to the existing literature. First, the evaluation of companies’ efficiency includes both a self and peer evaluation. Because the water industry consists of heterogeneous companies whose efficiency could be influenced by production costs and other environmental characteristics, we used cross-efficiency techniques. Moreover, the inclusion of GHG emissions as an undesirable output in the water production process allows us to measure both economic and environmental performance. This is a novel approach because, to the best of our knowledge, no prior studies have been conducted of the English and Welsh water industry that analyze companies’ eco-efficiency using cross-efficiency DEA techniques. The main differences between the proposed method and the traditional approaches used to measure eco-efficiency in the presence of undesirable outputs are as follows. First, unlike parametric approaches such as stochastic frontier analysis (SFA) (see for instance, [22]) our proposed approach does not make any assumptions regarding the functional form for the underlying production technology. Moreover, traditional non-parametric approaches (e.g., [23,24,25]) do not impose any weight restrictions on undesirable outputs. As a result, the weights might take zero values, which means that not all observations will be included in the calculation of efficiency. Our proposed approach overcomes this limitation by ensuring that weights take positive values. Furthermore, traditional DEA methods [26,27] depend on the self-evaluation of units by not taking into account the efficiency of other units. In contrast, our proposed approach uses cross-efficiency techniques by including both self and peer evaluation. To ensure that our efficiency scores are robust, we take into account the ranking priority of units under evaluation. Furthermore, we linked the results with the regulatory cycle to discuss the impact of regulation on companies’ eco-performance. Some policy implications are finally discussed.

## 2. Methods

**Figure 1:**
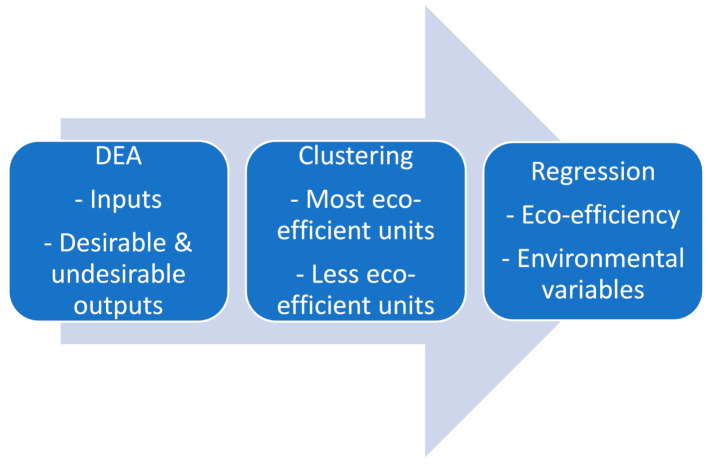
Eco-performance measurement of water utilities in our study.

In this section we discuss the methodology used to assess the eco-efficiency of several water utilities in England and Wales. Section 2.1 presents the DEA techniques to measure the relative eco-efficiency. The next section employs cluster techniques to group companies based on their eco-efficiency scores. Section 2.3 presents the econometric methods used to quantify the impact of several environmental variables on companies’ eco-efficiency. Figure 1 shows a visual presentation of the methodology used in this study.

### 2.1. Eco-Efficiency Assessment

This section discusses the methodology used to evaluate the eco-efficiency of several water utilities in England and Wales after taking into account undesirable outputs such as GHG emissions in the production process. Following the approach of Liu et al. [15], we ran several DEA models to assess water companies’ eco-efficiency. The first model was the traditional DEA model developed by Charnes et al. [11], which is a model for self-evaluation of DMUs. The limitation of this model is that weights might not be optimal, so efficiency (eco-efficiency) scores might be overestimated [18,28,29]. To overcome this limitation, we used cross-efficiency techniques, namely, both a self and peer evaluation technique [18]. However, the main limitation of this technique is that the weights might not be unique [16]. To overcome this issue, we ran a third DEA model, which takes into account DMUs’ best ranking positions and the efficiency of the other DMUs is minimized when the efficiency of each DMU is maximized [15].

Let’s assume that there are *k* DMUs (or water companies) (*k* = 1, …, *K*) that employ a set of *l* inputs, *x_ik_*(*i* = 1 … *l*) to produce a set of *m* desirable outputs, *y_rk_*(*r* = 1 … *m*) and *n* undesirable outputs, *y_bk_*(*b* = 1 … *p*). The traditional DEA model developed by Charnes et al. [11] is defined as follows:

$$\varphi_{k}=MAX\sum_{r=1}^{m}\mu_{rk}y_{rk}+\sum_{b=1}^{p}\varpi_{bk}y_{bk}\;k=1,2,\ldots ,K$$

s.t.:

$$\sum_{i=1}^{l}\nu_{ik}x_{ik}=1$$

$$\sum_{r=1}^{m}\mu_{rk}y_{rk}+\sum_{b=1}^{p}\varpi_{bk}y_{bk}-\sum_{i=1}^{l}\nu_{ik}x_{ik}\leq 0\;k=1,2,\ldots K$$

$$\mu_{rk}\geq 0\;r=1,2,\ldots m$$

$$\varpi_{bk}\geq 0\;b=1,2,\ldots p$$

(1) $$\nu_{ik}\geq 0\;i=1,2,\ldots l$$

where $u_{rk},{\bar{\omega }}_{bk},v_{ik}$ are the weights for the desirable outputs, undesirable outputs, and inputs, respectively. Model (1) is the first model that was used for the analysis of the eco-efficiency scores of water utilities. However, this model does not have any constraints regarding the weights of desirable and undesirable outputs, which means that there might be cases where the weights of desirable or undesirable outputs could be zero. Thus, the objective value φ will not include all weights for evaluation. To overcome this issue, Liu et al. [15] proposed constraints in the weights for desirable and undesirable outputs:

$$\Phi_{k}=MAX\sum_{r=1}^{m}\mu_{rk}y_{rk}+\sum_{b=1}^{p}\varpi_{bk}y_{bk}\;k=1,2,\ldots ,K$$

s.t.:

$$\sum_{i=1}^{l}\nu_{ik}x_{ik}=1$$

$$\sum_{r=1}^{m}\mu_{rk}y_{rk}+\sum_{b=1}^{p}\varpi_{bk}y_{bk}-\sum_{i=1}^{l}\nu_{ik}x_{ik}\leq 0\;k=1,2,\ldots .K$$

$$\sum_{r=1}^{m}\mu_{rk}y_{rk}\geq \Phi_{k}$$

$$\sum_{b=1}^{p}\varpi_{bk}y_{bk}\geq \Phi_{k}$$

$$\mu_{rk}\geq 0\;r=1,2,\ldots m$$

$$\varpi_{bk}\geq 0\;b=1,2,\ldots p$$

$$\nu_{ik}\geq 0\;i=1,2,\ldots l$$

(2) $$\nu_{ik}\geq 0\;i=1,2,\ldots l$$

where $\Phi_{{}_{k}}^{*}$ is a positive value for each water company k and its optimal value, $\Phi_{{}_{k}}^{*}$ is then used in the following model [15]:

$$\gamma_{k}=MAX\sum_{r=1}^{m}\mu_{rk}y_{rk}+\sum_{b=1}^{p}\varpi_{bk}y_{bk}\;k=1,2,\ldots ,K$$

s.t.:

$$\sum_{i=1}^{l}\nu_{ik}x_{ik}=1$$

$$\sum_{r=1}^{m}\mu_{rk}y_{rk}+\sum_{b=1}^{p}\varpi_{bk}y_{bk}-\sum_{i=1}^{l}\nu_{ik}x_{ik}\leq 0\;k=1,2,\ldots .K$$

$$\sum_{r=1}^{m}\mu_{rk}y_{rk}\geq \alpha *\Phi_{k}^{*}$$

$$\sum_{b=1}^{p}\varpi_{bk}y_{bk}\geq \beta *\Phi_{k}^{*}$$

$$\mu_{rk}\geq 0\;r=1,2,\ldots m$$

$$\varpi_{bk}\geq 0\;b=1,2,\ldots p$$

(3) $$\nu_{ik}\geq 0\;i=1,2,\ldots l$$

Model (3) prevents the weights of desirable and undesirable outputs from being zero and, therefore, they are all included in the evaluation. Parameters α and β denote the lower bounds that the weights of desirable and undesirable outputs are permitted to take in the evaluation [15]. In our study, α and β were set to 0.2, which is consistent with the study by Liu et al. [15]. Model (3) is the second model that was used to estimate the eco-efficiency scores of water utilities. After running Model (3), the eco-efficiency scores were calculated based on cross-efficiency techniques, i.e., self and peer evaluation of water companies [21]. The cross-efficiency score of each water company k was derived as follows:

(4) $$\gamma_{k}^{*}=\frac{1}{K}\sum_{k=1}^{K}\frac{\sum_{r=1}^{m}\mu_{rk}y_{rk}+\sum_{b=1}^{p}\varpi_{bk}y_{bk}}{\sum_{i=1}^{l}\nu_{ik}x_{ik}}$$

Because the optimal solution of Model (3) is not unique, water companies are eco-efficient or inefficient based on different optimal solutions, and thus take different ranking places. To overcome this issue, Liu et al. [15] proposed the ranking priority model where it identifies the best ranking position of a firm using its own optimal weights. The ranking priority model takes the following form:

$$\delta_{k}^{*}=MIN\sum_{k=1}^{K}\delta_{k}\;k=1,2,\ldots ,K$$

s.t.:

$$\sum_{i=1}^{l}\nu_{ik}x_{ik}=1$$

$$\sum_{r=1}^{m}\mu_{rk}y_{rk}+\sum_{b=1}^{p}\varpi_{bk}y_{bk}=\delta_{k}$$

$$\sum_{r=1}^{m}\mu_{rk}y_{rk}+\sum_{b=1}^{p}\varpi_{bk}y_{bk}-\sum_{i=1}^{l}\nu_{ik}x_{ik}\leq 0\;k=1,2,\ldots .K$$

$$\gamma_{k}*\sum_{i=1}^{l}\nu_{ik}x_{ik}-\sum_{r=1}^{m}\mu_{rk}y_{rk}-\sum_{b=1}^{p}\varpi_{bk}y_{bk}+r_{k}=0\;k=1,2,\ldots K$$

$$r_{k}\leq P*\delta_{k}\;k=1,2,\ldots ,K$$

$$\sum_{r=1}^{m}\mu_{rk}y_{rk}\geq \alpha *\Phi_{k}^{*}$$

$$\sum_{b=1}^{p}\varpi_{bk}y_{bk}\geq \beta *\Phi_{k}^{*}$$

$$r_{k}\;free\;k=1,2,\ldots ,K$$

$$\delta_{k}\in (0,1)\;k=1,2,\ldots ,K$$

$$\mu_{rk}\geq 0\;r=1,2,\ldots m$$

$$\varpi_{bk}\geq 0\;b=1,2,\ldots p$$

(5) $$\nu_{ik}\geq 0\;i=1,2,\ldots l$$

where $\gamma_{k}$ is the eco-efficiency score of each firm (water company) k estimated from Model (3) and P is a large positive value. To ensure that the ranking place and efficiency score is optimal, Liu et al. [15] developed the following model where the eco-efficiency of the other firms is minimized when the eco-efficiency of each firm is maximized [20]:

$$MAX\sum_{k=1}^{K}z_{k}\;k=1,2,\ldots ,K$$

s.t.:

$$\sum_{i=1}^{l}\nu_{ik}x_{ik}=1$$

$$\sum_{r=1}^{m}\mu_{rk}y_{rk}+\sum_{b=1}^{p}\varpi_{bk}y_{bk}=\delta_{k}$$

$$\sum_{r=1}^{m}\mu_{rk}y_{rk}+\sum_{b=1}^{p}\varpi_{bk}y_{bk}-\sum_{i=1}^{l}\nu_{ik}x_{ik}\leq 0\;k=1,2,\ldots .K$$

$$\gamma_{k}*\sum_{i=1}^{l}\nu_{ik}x_{ik}-\sum_{r=1}^{m}\mu_{rk}y_{rk}-\sum_{b=1}^{p}\varpi_{bk}y_{bk}+r_{k}=0\;k=1,2,\ldots K$$

$$r_{k}\leq P*\delta_{k}\;k=1,2,\ldots ,K$$

$$\delta_{k}^{*}=\sum_{k=1}^{K}\delta_{k}$$

$$\sum_{r=1}^{m}\mu_{rk}y_{rk}\geq \alpha *\Phi_{k}^{*}$$

$$\sum_{b=1}^{p}\varpi_{bk}y_{bk}\geq \beta *\Phi_{k}^{*}$$

$$z_{k}\geq 0$$

$$r_{k}\;free\;k=1,2,\ldots ,K$$

$$\delta_{k}\in (0,1)\;k=1,2,\ldots ,K$$

$$\mu_{rk}\geq 0\;r=1,2,\ldots m$$

$$\varpi_{bk}\geq 0\;b=1,2,\ldots p$$

(6) $$\nu_{ik}\geq 0\;i=1,2,\ldots l$$

where $\delta_{k}^{*}$ is the optimal value obtained from the ranking priority in Model (5). Model (6) was the third model used for the estimation of the eco-efficiency of water utilities. After obtaining a set of optimal weights for desirable and undesirable outputs and inputs $\left(u_{rk}^{*},{\bar{\omega }}_{bk}^{*},v_{ik}^{*}\right)$ from Model (6), then the cross-eco-efficiency score of each firm (water company) k can be calculated as follows:

(7) $$\mu_{k}^{*}=\frac{1}{K}\sum_{k=1}^{K}\frac{\sum_{r=1}^{m}\mu_{rk}^{*}y_{rk}+\sum_{b=1}^{p}\varpi_{bk}^{*}y_{bk}}{\sum_{i=1}^{l}\nu_{ik}^{*}x_{ik}}$$

### 2.2. Cluster Analysis

To better understand the eco-performance of water companies, we employed cluster analysis techniques to classify companies into groups based on their eco-efficiency scores. Cluster analysis has been widely used to deal with environmental issues [30,31]. The idea of cluster analysis is that objects are grouped together based on similar characteristics [32]. In the efficiency analysis framework, there were several studies that integrated cluster analysis and DEA techniques to measure firms’ efficiency and then group them based on their efficiency [32,33,34,35,36]. There are several approaches for clustering objects into homogeneous groups, such as k-means and hierarchical clustering (for an overview of these methods please see [37]). The main advantage of hierarchical clustering over the aforementioned approaches is its robustness. In contrast with k-means, it does not make any assumptions about the variance of the variables, or the amount of data in each cluster or the clusters. In addition, hierarchical clustering is less sensitive to outliers than other clustering methods [37]. Thus, our study used the hierarchical cluster technique to classify water companies based on their eco-efficiency scores. 

The hierarchical cluster algorithm is an iterative process [31]. Initially, every object (firm/data point) is considered as a cluster, i.e., there are as many clusters as data points. Then the two most similar clusters are merged (linkage process). The merging process continues until all of the data belong to a single cluster [31]. In this article, Ward’s linkage criterion was used to determine which clusters will be merged following past studies (see for instance [31,38]). Therefore, the minimum increase in the new cluster’s variance (Ward’s criterion) was used to determine the clusters that will be merged ([31,38,39]). 

### 2.3. Analysis of Eco-Efficiency Drivers

The final step of our analysis was to understand how several environmental variables might impact the eco-efficiency of water companies. To do this, we employed econometric techniques in which the dependent variable was the eco-efficiency score and examples of several explanatory variables could include population density and abstraction of water from rivers (see next section for more details). Because the eco-efficiency score is a truncated variable with values between zero and one, Tobit regression was used to derive robust estimates. Several studies in the past used Tobit analysis to understand the drivers of inefficiency in the production process (see for instance [4,12,40,41,42,43,44,45,46]). The model employed was defined as follows:

(8) $$\mu_{k,t}^{*}=\pi_{0}+\pi_{k}\zeta_{k,t}^{\prime }+\;\tau_{k}+\varepsilon_{k,t}$$

where $\mu_{k,t}^{*}$ is the cross eco-efficiency score of each water company k at any time t obtained from Equation (7), $\pi_{0}$ is the intercept term, $\zeta_{k}^{\prime }$ is a set of environmental variables and *π*_k_ are its related parameters to be estimated, $\tau_{k}$ denotes firm-specific unobserved heterogeneity (e.g., managerial inability), and $\varepsilon_{k,t}$ is the error term which fulfils the normal distribution, $N\left(0,\sigma^{2}\right)$.

## 3. Case Study Description

The empirical approach conducted in this study focused on the water services provided by Water and Sewerage Companies (WaSCs) and Water Only Companies (WoCs) in England and Wales during the years 2013–2018. Being natural monopolies, the Water Services Regulation Authority (Ofwat), which is the economic regulator, was set up to monitor the economic and environmental performance of water companies. This is done by approving water companies’ business plans and setting revenue allowances (price caps) every five years. 

To determine the eco-efficiency of the water companies we used the following inputs and desirable and undesirable outputs. The first input was defined as the energy expenditure (cost) for the provision of water services measured in millions of pounds each year [4,47]. The second input was defined as other water expenditure (cost) derived as the difference between water operating expenditure and energy expenditure [48,49]. Other water expenditure was also measured in millions of pounds each year. Three desirable outputs were selected for the purposes of our study. The first desirable output was the volume of water delivered measured in thousands of cubic meters per year [13,50,51]. The second desirable output was the number of water-connected properties measured in thousands per year [48,52]. The third desirable output was the length of water mains measured in thousands of kilometers per year [53,54]. The undesirable output was the GHG emissions from the supply of water services. It was expressed in tonnes of carbon dioxide equivalent, CO_2_ equivalent (CO_2eq_) per year [4,13,51,55], and was measured based on the UK Government Environmental Reporting Guidelines [4,56]. GHG emissions are related to company’s activities to abstract, treat, and supply water to its customers [57,58]. 

To better understand the drivers of eco-inefficiency in the production process, we used the following environmental variables in our estimation. These were selected to capture the different operating characteristics of the companies, such as topography, treatment complexity, and population density [4]. Thus, the percentages of water taken from boreholes and rivers, and average pumping head capture topography [49,50,59,60]. The more water is abstracted from rivers or boreholes, the higher the pumping (energy) requirements could be. This could lead to higher GHG emissions and lower inefficiency. Two variables were used to capture the water treatment complexity. The first was the percentage of water that receives an advanced level of water treatment (for more detail see [4,61,62,63,64]). The second was the number of treatment works when water is taken from surface water resources [62]. Finally, population density was defined as the ratio of population to the length of water mains [65,66]. Table 1 depicts the descriptive statistics of the variables used in the study. 

## 4. Results

This section discusses the results obtained from the use of different techniques to analyze the eco-efficiency of water utilities. Section 4.1 presents the findings from the implementation of different DEA models to evaluate relative efficiency and the use of clustering techniques to analyze eco-efficiency scores. Section 4.2 presents the results from regressing the eco-efficiency scores on several environmental variables and determines their impact.

### 4.1. Eco-Efficiency Estimation

Average eco-efficiency scores for WaSCs and WoCs estimated based on Models (1) and (3) are first presented. Both models refer to the traditional DEA model developed by [11] ((Charnes, Cooper, Rhodes (CCR) model). Model (1) allows for weights to be flexible (take zero values) whereas Model (3) prevent weights from taking zero values. The eco-efficiency scores were calculated based on the self-evaluation framework and a summary is reported in Table 2. The results indicate that under Model (1), 37 out of 102 (36.3%) observations reported zero values for their weighted sum of outputs. This means that these observations were not taken into account in the evaluation. Moreover, 24 out of 37 (64.9%) observations reported zero values for the undesirable output. This means that only desirable outputs were considered in the evaluation. In contrast, under Model (3), as expected, there were no observations whose outputs weights had zero values. This means that both desirable and undesirable outputs were included in the evaluation. Thus, Model (3) is more appropriate than Model (1) to assess the eco-efficiency of water utilities.

It was found that during the years 2013–2018 on average WaSCs were 0.918 carbon efficient, which means that energy, other costs, and GHG emissions could be reduced by 8.2% to maintain the same level of output. As shown in Table 3, all WaSCs reported high levels of eco-efficiency, with values ranging from 0.875 to 1. The average WoC reported a lower level of eco-efficiency than the average WaSC. It was found that on average WoCs reported an eco-efficiency of 0.892. This suggests that the average WoC could reduce costs and GHG emissions by 11.8% to improve its eco-efficiency. 

The eco-efficiency scores presented in Table 3 refer to the self-evaluation only of the water companies. Table 4 presents the eco-efficiency scores that include both self and peer evaluation. These are the cross-efficiency scores derived after running DEA Models (3) and (5). It was found that both models reported lower eco-efficiency scores than the those evaluated using the self-evaluation framework. On average, the English and Welsh water industry needed to reduce GHG emissions and costs by 23% to achieve higher carbon efficiencies. Moreover, the cross-efficiency scores from both models can be employed to rank water companies in different positions [15]. However, the two techniques reported different results in ranking. For instance, under Model (3) the most eco-efficient company was WoC4, whereas under Model (5) it was WaSC1. Several other companies changed their ranking places under these two approaches. For instance, WaSC8 ranked 12th under Model (3) and 10th under Model (5). Changes in ranking places were reported for WoC1, from 10th under Model (3) to 12th under Model (5). These differences in rankings along with efficiencies are due to the assumptions underlining each model. Moreover, unlike Model (3), Model (5) guarantees the uniqueness of the optimal weights of each firm [15]. Thus, Model (5) is considered more appropriate than Model (3), and the eco-efficiency of WaSCs and WoCs is further discussed based on this approach. This finding illustrates the importance of using robust and reliable methods to evaluate the performance of water companies, especially when results are used for regulatory purposes.

**Figure 2:**
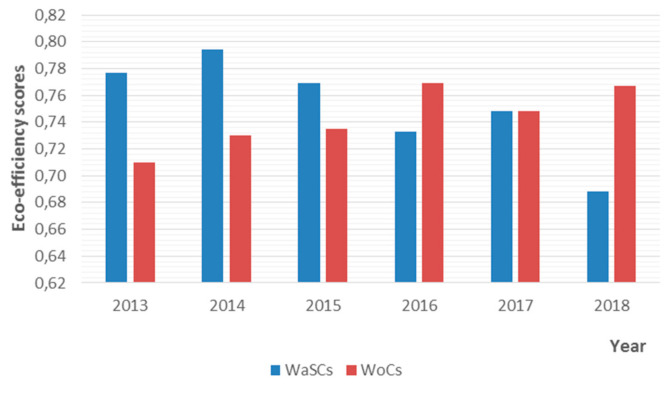
Evolution of eco-efficiency over time for water and sewerage companies (WaSCs) and water only companies (WoCs) estimated using Model (5).

Figure 2 reports the cross eco-efficiency scores for WaSCs and WoCs during the years 2013–2018 based on DEA Model (5). We split the period of study into two sub-periods, 2013–2015 and 2016–2018, to link the results with the regulatory cycle. The findings indicate that the water companies did not perform well in reducing their carbon emissions, as shown by their eco-efficiency scores. It was found that on average WaSCs and WoCs reported an efficiency score of 0.752 and 0.743, respectively. This means that WaSC and WoC could generate the same output with 24.8% and 25.7%, respectively, of costs and CO_2eq_ emissions used if they were producing on the eco-efficient frontier. We note that the average WaSC’s eco-efficiency deteriorated over time, whereas the average WoC’s efficiency followed an upward trend. The differences in eco-efficiency scores between WaSCs and WoCs could be attributed to the expenditure of water companies’ themselves. Another reason could be abstracting water from different resources, which might require different levels of treatment, thus eventually increasing treatment costs and CO_2eq_ emissions [4].

During the years 2013–2015, which is the period covered by the 2009 price review, the regulator introduced several financial incentives to boost companies’ economic and environmental performance. These included a rolling mechanism by which the water companies were permitted to maintain any savings in operating expenditure regardless of the year occurred [60]. Other schemes included the Service Incentive Mechanism (SIM) to financially reward companies who improved quality of service to customers. WaSC’s efficiency peaked in 2014 but eventually decreased the following year. It is shown that during the years 2013–2015, WaSC’s eco-efficiency was decreasing at a rate of 0.4% per year. In contrast, WoC’s eco-efficiency increased by 3.5% on average, from 0.710 in 2013 to 0.735 in 2015. Thus, WaSCs need to better manage their daily operations by moving to a more efficient allocation of their resources to improve their cost and environmental performance. 

The next sub-period (2016–2018) refers to the period covered by the 2014 price review. The water regulator introduced several common performance indicators to measure the economic and environmental performance of water companies. When companies delivered the promised service levels to their customers, they received financial rewards; otherwise, financial penalties were imposed [60]. The results showed that the downward trend in WaSCs’ eco-efficiency continued. This might be attributed to increases in energy and other costs which might have led to higher levels of CO_2eq_ emitted in the atmosphere. It is noted that, compared to 2013, the average WaSC’s eco-efficiency reduced from 0.777 in 2013 to 0.688 in 2018. In contrast, the average WoC’s eco-efficiency increased to the level of 0.767, an 8% improvement relative to 2013. Overall, the results indicate that both WaSCs and WoCs need to achieve savings in their production costs and curtail their GHG emissions. This is particularly evident for WaSCs who showed a deterioration in their eco-performance over time. Water companies could employ new practices and technologies that could help them reduce treatment costs (e.g., energy and chemical) and carbon emissions. Another example could be the use of energy from renewable sources, which could reduce the cost of treatment and the level of emissions released in the atmosphere from the provisions of water services.

Because cross-eco-efficiency scores differ among WaSCs and WoCs and across years (Table 4 and Figure 1), we employed cluster analysis techniques to classify water companies into similar groups based on their eco-efficiency scores across all years of our sample. The results are reported in Table 5. The clustering analysis revealed two groups. The first group comprised the most eco-efficient companies, and the second group the less eco-efficient companies. The most eco-efficient group had five WaSCs and three WoCs. During the whole period of study, the mean eco-efficiency was 0.831, which means that on average the companies needed to reduce costs and CO_2eq_ emissions by 16.9% to generate the same level of output. Eco-efficiency improvements for this group ranged between 11.0% and 23.3%. 

The less eco-efficient group consisted of five WaSCs and four WoCs. On average this group showed a low eco-efficiency score across all years. It was found that a decrease in both costs and CO_2eq_ emissions by 32.5% is required to provide the same level of output. Thus, the less eco-efficient water companies need to make substantial improvements to improve both economic and environmental performance to catch up with the most eco-efficient companies in the industry. For instance, the worst eco-efficient company needs to reduce costs and CO_2eq_ by 40% to be eco-efficient. This group of water companies needs to serve more customers and deliver high volumes of water. In contrast, the most eco-efficient group includes water companies with lower volume of water to treat and distribute because the number of customers is lower. We further note that, for this group of water companies, the density levels and energy requirements to abstract water from different resources are lower than the those from the less eco-efficient group. Therefore, it seems that the eco-efficiency of the companies might be influenced not only by production costs but also other operating characteristics. 

### 4.2. Drivers of Eco-Efficiency 

Table 6 presents the results from Tobit regression, which allowed us to quantify the impact of the operating and environmental variables on water companies’ eco-efficiency. It is illustrated that water treatment complexity, number of treatment works for surface water, population density, and average pumping head had a statistically significant impact on eco-efficiency. It was found that the higher the level of water treatment, the higher the costs to treat and thus the higher the level of GHG emissions released to the atmosphere. Keeping things equal, one unit increase in the level of water treatment might lead to a 0.571 unit decrease in water companies’ eco-efficiency. Similarly, the higher the number of treatment works for surface water, the lower the eco-efficiency of the water companies could be. This means that surface water might require high levels of treatment before it is distributed to end users. As a result, higher treatment costs could lead to higher GHG emissions and eventually lower eco-efficiency. This finding evidences the importance of adopting policies at the watershed level. Population density is an important driver of water companies’ eco-efficiency. The more densely populated the area, lower the reported eco-efficiency of water companies. Technically speaking, a one unit increase in population density could lead to a reduction in average eco-efficiency by 0.291 units. Finally, the more water is abstracted, the higher the energy requirements to pump water to the treatment plants and customers. Thus, higher average pumping head could lead to higher costs and GHG emissions and, eventually, lower eco-efficiency. 

The results of our study could be of great interest to stakeholders for the following reasons. First, we provide a methodology to identify the eco-efficiency of the water companies over time. We also highlight the importance of selecting a methodology that can provide robust and accurate eco-efficiency estimates. Our study showed that the water companies in England and Wales need to make more effort to improve the way they run their operations. Water companies should move to a more efficient allocation of resources that could allow them to be more economic and environmentally efficient. For instance, the adoption of new technologies to reduce costs during the treatment process and curtail carbon emissions could be of great importance. Finally, the methodology applied in this study shows that there might be other factors, such as water treatment complexity and population density, that could influence companies’ eco-efficiency. These aspects should be taken into account in the decision-making process for a sustainable, healthy, and efficient urban cycle.

## 5. Conclusions

In the light of climate change and increased population growth, water companies need to make efforts to improve not only their economic performance but also their environmental performance. Improving the eco-efficiency in the water production process could lead to lower economic costs and, eventually, lower GHG emissions released in the atmosphere. This is particularly evident in the UK water industry, which seeks to move to a carbon-free industry by 2050. 

This study provides evidence on the eco-efficiency of the water companies in England and Wales during the years 2013–2018. We analyzed their eco-efficiency scores based on a robust DEA model using cross-efficiency techniques. This technique considers both the self and peer evaluation of all water companies. We further analyzed companies’ eco-performance using cluster techniques that allowed us to assemble water companies in homogeneous groups based on their eco-efficiency scores. Finally, we obtained a better insight into the drivers of companies’ eco-efficiency using econometric techniques. The results can be summarized as follows. First, it is concluded that a model that took into account the ranking preferences and competitive environment of firms could lead to robust eco-efficiency estimates. Moreover, it was found that on average the English and Welsh water industry needed to manage better its managerial practices to improve eco-efficiency. In particular, the average WaSC needed to further reduce its costs and CO_2eq_ emissions by 24.8% to generate the same level of output. The average WoC could produce the same output with 25.7%, of costs and CO_2eq_ emissions used if they were producing on the frontier. The findings showed that there was a downward trend in WaSC’s eco-efficiency, whereas the opposite was true for WoCs. It appears that the price review did not have a major impact on water companies’ performance because additional savings in costs and GHG emissions should be pursued in the following years. Our cluster analysis showed that during the whole period of study, two types of groups exist in the industry, i.e., the most eco-efficient group and the less eco-efficient group. The latter group needed to reduce its costs and carbon emissions by 32.5% on average to improve performance relative to the most eco-efficient group. It seems that water companies’ eco-efficiency is influenced not only by production costs but also other operating characteristics, such as population density and average pumping head. Advanced levels of water treatment, high energy requirements to distribute water to customers, and densely populated areas could increase costs and lead to higher carbon emissions. It was found that keeping things equal, a one unit increase in the level of water treatment and population density could lead to 0.571 and 0.291 unit decreases in average water companies’ efficiency, respectively. 

We note that this methodology included GHG emissions from the supply of water services. This methodology can be extended to measure the eco-efficiency of both water and wastewater services, therefore covering all stages of the water and wastewater supply chain. This would require information regarding GHG emissions from the provision of wastewater services. Moreover, this methodology could apply to any other sector of the economy that contributes to the release of carbon emissions in the atmosphere, such as the agriculture and transport sectors. It can also be extended at a larger scale if the researcher is interested in measuring the carbon (or energy) efficiency of countries using macro-level data. We finally note that this methodology could also be used to measure the efficiency of other undesirable outputs, such as water leakage and unplanned interruptions, or any other pollutants, such as air pollution, by taking into account socio-economic data from human activities such as energy use and transport. Thus, these applications could provide insightful contributions to a more sustainable, efficient, and healthy economy and society. 

Overall, the following conclusions could be drawn from a policy perspective. First, improving the eco-efficiency in the water production process by reducing energy costs and GHG emissions is among the challenges that water companies will be faced with in the future. Thus, it is important for them to know how eco-efficient they have been over time and what impacts inefficiency. The adoption of new technologies to reduce treatment costs or the use of renewable energy could be of great importance to reduce costs and carbon emissions. Eco-inefficiency could be attributed not only to energy costs and carbon emissions, but also other characteristics related to topography and density of water companies. Our study showed that high water treatment complexity and densely populated areas could be additional factors that impact eco-efficiency and should be incorporated in the business decision-making process.

## Figures and Tables

**Table 1:** Descriptive statistics of the variables used.

Variables	Unit of Measurement	Mean	Std. Dev.	Min.	Max.
Volume of water delivered	000 s m^3^/year	713	555	56	2169
Number of water connected properties	000 s/year	1499	1125	124	3826
Length of water mains	000 s km	20,451	14,099	2024	47,817
Greenhouse gas emissions	tonCO_2eq_/year	82,845	69,062	4542	275,900
Energy costs	₤m/year	20	15	2	60
Other costs	₤m/year	93	79	8	332
Water taken from rivers	%	22.9	20.9	0.1	73.2
Water taken from boreholes	%	40.0	30.8	3.5	92.1
Surface water treatment works	nr	16	15	1	54
Water receiving high levels of treatment	%	93.1	5.4	81.0	100.0
Average pumping head	nr	147	44	65	256
Population density	000 s/km	0.167	0.048	0.107	0.316
Observations		108			

Energy and other costs are expressed in 2018 prices.

**Table 2:** Summary of pooled statistics of eco-efficiency estimations.

Estimates	Model (1)			Model (3)		
	WaSCs	WoCs	All Water Companies	WaSCs	WoCs	All Water Companies
Average eco-efficiency	0.930	0.896	0.916	0.918	0.892	0.907
Number of DMUs whose sum of weights of desirable outputs takes zero values	8	5	13	0	0	0
Number of DMUs whose sum of weights of undesirable outputs takes zero values	20	4	24	0	0	0

**Table 3:** Eco-efficiency of water companies estimated using Model (3).

Water Company	Eco-Efficiency Score	Sum of Weights of Desirable Outputs	Sum of Weights of Undesirable Output
WaSC1	0.978	0.440	0.538
WaSC2	0.795	0.650	0.145
WaSC3	0.936	0.714	0.222
WaSC4	0.875	0.375	0.500
WaSC5	0.952	0.640	0.312
WaSC6	0.930	0.915	0.015
WaSC7	0.841	0.645	0.196
WaSC8	0.930	0.856	0.074
WaSC9	1.000	0.100	0.900
WaSC10	0.941	0.636	0.305
WoC1	0.865	0.390	0.475
WoC2	0.776	0.423	0.353
WoC3	0.797	0.416	0.381
WoC4	1.000	0.590	0.410
WoC5	0.885	0.786	0.100
WoC6	0.968	0.349	0.619
WoC7	0.952	0.231	0.721
Average WaSC	0.918	0.597	0.321
Average WoC	0.892	0.455	0.437
Average	0.907	0.539	0.369

**Table 4:** Cross-eco-efficiency scores and ranking of water companies.

Water Company	Model (3)		Model (5)	
	Eco-Efficiency Score ($\gamma_{k}^{*}$)	Rank	Eco-Efficiency Score ($\mu_{k}^{*}$)	Rank
WaSC1	0.905	3	0.890	1
WaSC2	0.670	15	0.639	15
WaSC3	0.790	8	0.767	8
WaSC4	0.764	9	0.744	9
WaSC5	0.870	4	0.835	4
WaSC6	0.601	17	0.618	16
WaSC7	0.648	16	0.591	17
WaSC8	0.708	12	0.723	10
WaSC9	0.907	2	0.882	3
WaSC10	0.851	5	0.826	5
WoC1	0.742	10	0.710	12
WoC2	0.692	13	0.667	14
WoC3	0.725	11	0.712	11
WoC4	0.910	1	0.885	2
WoC5	0.684	14	0.668	13
WoC6	0.832	6	0.783	6
WoC7	0.820	7	0.777	7
Average WaSC	0.772		0.752	
Average WoC	0.772		0.743	
Average	0.772		0.748	

**Table 5:** Cluster analysis of eco-efficiency scores.

Clusters	Average	Min	Max	Water Companies
Cluster I	0.831	0.767	0.890	WaSC1, WaSC3, WaSC5, WaSC9, WaSC10, WoC14, WoC16, WoC17
Cluster II	0.675	0.591	0.744	WaSC2, WaSC4, WaSC6, WaSC7, WaSC8, WoC11, WoC12, WoC13, WoC15

**Table 6:** Estimates of Tobit regression: variables affecting eco-efficiency scores.

Variables	Coef.	Std. Err.	Z-Stat.	*p*-Value
Constant	−0.156	0.383	−0.410	0.684
% of water taken from boreholes	0.084	0.070	1.190	0.234
Water treatment complexity	−0.571	0.340	***−1.680***	0.093
% of water taken from rivers	0.041	0.077	0.540	0.592
Number of SW treatment works	−0.003	0.002	**−2.000**	0.046
Population density	−0.293	0.075	**−3.920**	<0.001
Average pumping head	−0.001	<0.001	**−2.690**	0.007
Year				
2014	0.021	0.023	0.950	0.343
2015	0.009	0.023	0.390	0.694
2016	0.003	0.023	0.150	0.881
2017	−0.007	0.023	−0.310	0.760
2018	−0.047	0.026	***−1.830***	0.067
Log-likelihood	120.05			
X^2^(11)	**24.95**			
Prob > X^2^(11)	0.009			

Eco-efficiency score is the dependent variable; bold coefficients are statistically significant from zero at the 5% level. Bold italic coefficients are statistically significant from zero at the 10% level.

## Data Availability

The data presented in this study are available on request from the corresponding author. The data are not publicly available due to legal and privacy issues.

## References

[1] Kumar A., Thanki A., Padhiyar H., Singh K.N., Pandey S., Yadav M., Yu Z.G. (2021). Greenhouse gases emission control in WWTS via potential operational strategies: A critical review. Chemosphere.

[2] Liu L., Sun X., Chen C., Zhao E. (2016). How will auctioning impact on the carbon emission abatement cost of electric power generation sector in China?. Appl. Energy.

[3] Madolo S.D., Telukdarie A., Kumar A. (2018). Energy–water and GHG nexus: A South African water industry case. Water Pract. Technol..

[4] Sala-Garrido R., Mocholi-Arce M., Molinos-Senante M., Maziotis A. (2021). Marginal abatement cost of carbon dioxide emissions in the provision of urban drinking water. Sustain. Prod. Consum..

[5] Kumar A., Yang T., Sharma M.P. (2019). Greenhouse gas measurement from Chinese freshwater bodies: A review. J. Clean. Prod..

[6] Wakeel M., Chen B., Hayat T., Alsaedi A., Ahmad B. (2018). Energy consumption for water use cycles in different countries: A review. Appl. Energy.

[7] Chen P.-C., Alvarado V., Hsu S.-C. (2018). Water energy nexus in city and hinter- lands: Multi-regional physical input-output analysis for Hong Kong and South China. Appl. Energy.

[8] Liao X., Zhao X., Liu W., Li R., Wang X., Wang W., Tillotson M.R. (2020). Comparing water footprint and water scarcity footprint of energy demand in China’s six megacities. Appl. Energy.

[9] Ananda J. (2018). Productivity implications of the water-energy-emissions nexus: An empirical analysis of the drinking water and wastewater sector. J. Clean. Prod..

[10] Suárez-Varela M., de los Ángeles García-Valiñas M., González-Gómez F., Picazo-Tadeo A.J. (2018). Ownership and Performance in Water Services Revisited: Does Private Management Really Outperform Public?. Water Resour. Manag..

[11] Charnes A., Cooper W.W., Rhodes E. (1978). Measuring the efficiency of decision making units. Eur. J. Oper. Res..

[12] Ananda J. (2019). Explaining the environmental efficiency of drinking water and wastewater utilities. Sustain. Prod. Consum..

[13] Ananda J., Hampf B. (2015). Measuring environmentally sensitive productivity growth: An application to the urban water sector. Ecol. Econ..

[14] Ding L., Yang Y., Wang W., Calin A.C. (2019). Regional carbon emission efficiency and its dynamic evolution in China: A novel cross efficiency-malmquist productivity index. J. Clean. Prod..

[15] Liu X., Chu J., Yin P., Sun J. (2017). DEA cross-efficiency evaluation considering undesirable output and ranking priority: A case study of eco-efficiency analysis of coal-fired power plants. J. Clean. Prod..

[16] Doyle J., Green R.H. (1994). Efficiency and Cross-Efficiency in DEA: Derivations, Meanings and Uses. J. Oper. Res. Soc..

[17] Doyle J.R., Green R.H. (1995). Cross-evaluation in DEA: Improving discrimination among DMUs. INFOR.

[18] Wang Y.-M., Chin K.-S. (2010). Some alternative models for DEA cross-efficiency evaluation, *Int*. J. Prod. Econ..

[19] Sexton T.R., Silkman R.H., Hogan A.J. (1986). Data envelopment analysis: Critique and extensions. New Dir. Program Eval..

[20] Cui Q., Li Y. (2020). A cross efficiency distinguishing method to explore the cooperation degree in dynamic airline environmental efficiency. Transp. Policy.

[21] Bevilacqua M., Ciarapica F.E., Mazzuto G., Paciarotti C. (2015). Efficiency assessment of blanching and deep-freezing systems through data envelopment analysis. Eng. Agric. Environ. Food.

[22] Kutlu L. (2020). Greenhouse Gas Emission Efficiencies of World Countries. Int. J. Environ. Res. Public Health.

[23] Molinos-Senante M., Hernandez-Sancho F., Mocholi-Arce M., Sala-Garrido R. (2014). Economic and environmental performance of wastewater treatment plants: Potential reductions in greenhouse gases emissions. Resour. Energy Econ..

[24] Mukherjee K. (2010). Measuring energy efficiency in the context of an emerging economy: The case of Indian manufacturing. Eur. J. Oper. Res..

[25] Vlontzos G., Niavis S., Manos B. (2014). A DEA approach for estimating the agricultural and environmental efficiency of EU countries. Renew. Sustain. Energy Rev..

[26] Färe R., Grosskopf S. (2004). Modeling undesirable factors in efficiency evaluation: Comment. Eur. J. Oper. Res..

[27] Zhou P., Ang B.W. (2008). Linear programming models for measuring economy-wide energy efficiency performance. Energy Policy.

[28] Liang L., Wu J., Cook W.D., Zhu J. (2008). Alternative secondary goals in DEA cross-efficiency evaluation, *J*. Clean. Prod..

[29] Wu J., Chu J., Sun J., Zhu Q., Liang L. (2016). Extended secondary goal models for weights selection in DEA cross-efficiency evaluation. Comput. Ind. Eng..

[30] Caballero S., Esclapez R., Galindo N., Mantilla E., Crespo J. (2012). Use of a passive sampling network for the determination of urban NO2 spatiotemporal variation. Atmos. Environ..

[31] Xie L., Chen C., Yu Y. (2019). Dynamic Assessment of Environmental Efficiency in Chinese Industry: A Multiple DEA Model with a Gini Criterion Approach. Sustainability.

[32] Bojnec S., Latruffe L. (2007). Measures of farm business efficiency. Ind. Manag. Data Syst..

[33] Sikka V., Luke R.D., Ozcan Y.A. (2009). The efficiency of hospital based clusters: Evaluating system performance using data envelopment analysis. Health Care Manag. Rev..

[34] Wu J., Liang L., Song M. (2010). Performance Based Clustering for Benchmarking of Container Ports: An Application of Dea and Cluster Analysis Technique. Int. J. Comput. Intell. Syst..

[35] Omrani H., Shafaat K., Emrouznejad A. (2018). An integrated fuzzy clustering cooperative game data envelopment analysis model with application in hospital efficiency, *Expert Syst*. Appl..

[36] Cinaroglou S. (2020). Integrated k-means clustering with data envelopment analysis of public hospital efficiency. Health Care Manag. Sci..

[37] Kaufman L., Rousseeuw P.J. (1990). Finding Groups in Data: An Introduction to Cluster Analysis.

[38] Gu F., Hall P., Nicholas J. (2016). Performance evaluation for composites based on recycled polypropylene using principal component analysis and cluster analysis. J. Clean. Prod..

[39] Suzuki R., Shimodaira H. (2006). Pvclust: An R package for assessing the uncertainty in hierarchical clustering. Bioinformatics.

[40] Byrnes J., Crase L., Dollery B., Villano R. (2010). The relative economic efficiency of urban water utilities in regional New South Wales and Victoria. Resour. Energy Econ..

[41] Guerrini A., Romano G., Leardini C., Martini M. (2015). The Effects of Operational and Environmental Variables on Efficiency of Danish Water and Wastewater Utilities. Water.

[42] Wang X., Han L., Yin L. (2017). Environmental Efficiency and Its Determinants for Manufacturing in China. Sustainability.

[43] Ding Z.Y., Jo G.S., Wang Y., Yeo G.T. (2015). The relative efficiency of container terminals in small and medium-sized ports in China. Asianj. Ship. Log..

[44] Luo J.H., Cui E., Ji J.H. (2013). Analysis on container ports efficiency and its influencing factors on two stage method of DEA-TOBIT. Sci. Technol. Manag. Res..

[45] Wang L., Zhou Z., Yang Y., Wu J. (2020). Green efficiency evaluation and improvement of Chinese ports: A cross-efficiency model. Transp. Res. Part D.

[46] Zhang J., Fang H., Peng B., Wang X., Fang S. (2016). Productivity Growth-Accounting for Undesirable Outputs and Its Influencing Factors: The Case of China. Sustainability.

[47] Stone and Webster Consultants (2004). Investigation into Evidence for Economies of Scale in the Water and Sewerage Industry in England and Wales.

[48] Molinos-Senante M., Porcher S., Maziotis A. (2017). Impact of Regulation on English and Welsh Water-Only Companies: An Input Distance Function Approach. Environ. Sci. Pollut. Res..

[49] Molinos-Senante M., Maziotis A. (2018). Assessing the influence of exogenous and quality of service variables on water companies’ performance using a true-fixed stochastic frontier approach. Urban Water J..

[50] Brea-Solis H., Perelman S., Saal D.S. (2017). Regulatory incentives to water losses reduction: The case of England and Wales. J. Prod. Anal..

[51] Molinos-Senante M., Guzman C. (2018). Reducing CO_2_ emissions from drinking water treatment plants: A shadow price approach. Appl. Energy.

[52] Saal D.S., Parker D., Weyman-Jones T. (2007). Determining the contribution of technical efficiency, and scale change to productivity growth in the privatized English and Welsh water and sewerage industry: 1985–2000. J. Product. Anal..

[53] Thanassoulis E. (2000). Use of data envelopment analysis in the regulation of UK water utilities: Water distribution. Eur. J. Oper. Res..

[54] Thanassoulis E. (2002). Comparative performance measurement in regulation: The case of English and Welsh sewerage services. J. Oper. Res. Soc..

[55] Molinos-Senante M., Hanley N., Sala-Garrido R. (2015). Measuring the CO2 shadow price for wastewater treatment: A directional distance function approach. Appl. Energy.

[56] HM Government (2019). Environmental Reporting Guidelines: Including Streamlined Energy and Carbon Reporting Guidance March 2019 (Updated Introduction and Chapters 1 and 2).

[57] Ofwat (2010). Preparing for the Future –Ofwat’s Climate Change Policy Statement.

[58] Ofwat (2010). Playing Our Part—Reducing Greenhouse Gas Emissions in the Water and Sewerage Sectors Supporting Information.

[59] Molinos-Senante M., Maziotis A. (2020). Drivers of productivity change in water companies: An empirical approach for England and Wales. Int. J. Water Resour. Dev..

[60] Villegas A., Molinos-Senante M., Maziotis A. (2019). Impact of environmental variables on the efficiency of water companies in England and Wales: A double-bootstrap approach. Environ. Sci. Pollut. Res..

[61] Ofwat (2018). Cost Assessment for PR19: A Consultation on Econometric Cost Modelling.

[62] Ofwat (2019). PR19 Final Determinations: Securing Cost Efficiency Technical Appendix.

[63] Ofwat (2019). PR19 Final Determinations: Supplementary Technical Appendix: Econometric Approach.

[64] Ofwat (2019). RAG 4.0—Guideline for the Table Definitions in the Annual Performance Report.

[65] Molinos-Senante M., Porcher S., Maziotis A. (2018). Productivity change and its drivers for the Chilean water companies: A comparison of full private and concessionary companies. J. Clean. Prod..

[66] Molinos-Senante M., Villegas A., Maziotis A. (2019). Are water tariffs sufficient incentives to reduce water leakages? An empirical approach for Chile. Util. Policy.

